# SubmitoLoc: Identification of mitochondrial sub cellular locations of proteins using support vector machine

**DOI:** 10.6026/97320630015863

**Published:** 2019-12-31

**Authors:** Varadharaju Nithya

**Affiliations:** 1 Department of Animal Health Management, Alagappa University, Karaikudi-630003, India

**Keywords:** SVM, sub mitochondrial, protein prediction

## Abstract

Mitochondria are important sub-cellular organelles in eukaryotes. Defects in mitochondrial system lead to a variety of disease. Therefore, detailed knowledge of mitochondrial proteome is vital to understand mitochondrial system and their function. Sequence databases contain large number of mitochondrial proteins but they are mostly not annotated. In this study, we developed a support vector machine approach, SubmitoLoc, to predict mitochondrial sub cellular locations of proteins based on various sequence derived properties. We evaluated the predictor using 10-fold cross validation. Our method achieved 88.56 % accuracy using all features. Average sensitivity and specificity for four-subclass prediction is 85.37% and 87.25% respectively. High prediction accuracy suggests that SubmitoLoc will be useful for researchers studying mitochondrial biology and drug discovery.

## Background

Mitochondria are essential sub-cellular organelles of eukaryotes [1]. The primary role of mitochondria is to synthesize ATP through electron transport chain and oxidative phosphorylation [2]. It consists of two membranes, the inner membrane and the outer membrane, and two aqueous compartments, the inter membrane space and the matrix. Most of the mitochondrial proteins are synthesized in the cytoplasm and then imported into mitochondria by protein machineries located in the mitochondrial membranes [2]. Mitochondria involve in several biological processes such as programmed cell death, calcium signaling, ionic homeostasis etc [3]. It has been shown that mutation in genes that ecocide mitochondrial proteins leads to various rare human diseases like Leber's hereditary optic neuropathy, Leigh syndrome, Mitochondrial myopathy, hearing loss, and diabetes mellitus [4]. Therefore, detailed knowledge of mitochondrial proteome and their functions in various sub mitochondrial locations is very important for designing mitochondrial disorder therapies.

Various sequence databases provide experimentally verified mitochondrial subcelluar locations of proteins, but this list is very small. Further, designing experiments to obtain subcelluar locations of all mitochondrial proteins is expensive and time-consuming. Hence, it is necessary to develop bioinformatics methods based on machine learning algorithms for identifying mitochondrial proteins and its subclasses. In past, various machine-learning algorithms have been developed for prediction of mitochondrial proteins, although most were not proposed solely for mitochondrial proteins. TargetP [5], PSORT [6], MitoFates [7], MITOPROT [8], TPpred3 [9] and Predotar [10] are some of the popular methods that use target peptide or cleavage site information to predict mitochondrial proteins. The major limitation of these methods is that not all proteins have signal peptides. MITOPRED, MitPred and PFMpred are some of the methods that use protein sequence information instead of signal peptides. MITOPRED uses pfam domain and amino acid composition [11]. MitPred method use both support vector machine and hidden Markov model for predicting mitochondrial proteins [12]. PFMpred method predicts mitochdrial proteins using PSSM profile and spilit amino acid composition [13]. Tan et al., 2007 reported mitochondrial protein prediction method based on genetic algorithm and SVM [14].

**Figure 1:**
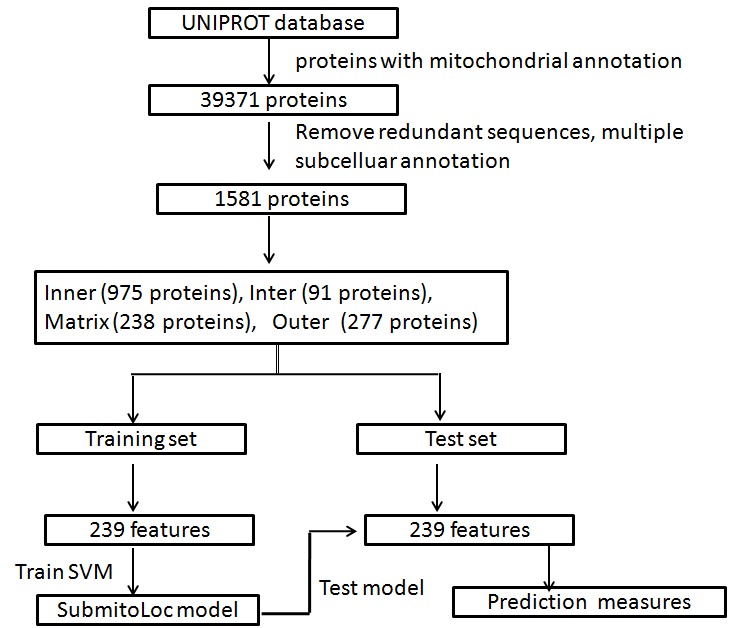
Flowchart representing various steps involved in SubmitoLoc method; Inner- inner membrane, inter-inter membrane space, outer - outer membrane space, matrix- mitochondrial matrix

Recently, some machine learning approaches for predicting protein submitochondrial locations have been proposed in the literature: Some of the methods are SUBmito [15], Gp-Loc [16], Predict_subMITO [17], TetraMito [18], Submitopred [19] and SubLoc [20]. Hoseini et al 2018 reported a method to predict protein sub mitochondrial locations using protein interaction networks [21]. Although several methods are available for the prediction of protein sub mitochondrial locations, most of these methods are limited to the prediction of three sub mitochondrial locations (3 compartments). Moreover, they are developed using a small dataset. Therefore, it is of interest to describe the identification of mitochondrial sub cellular locations of proteins from sequence derived properties using Support Vector Machine (SVM) abbreviated as SubmitoLoc in this report. Various steps involved in SubmitoLoc prediction system are summarized in (Figure 1).

## Methodology

### Dataset:

A set of 39371 proteins sequences was extracted from the SWISS-PROT database based on mitochondrial sub-cellular localization annotations in the comments block [21]. We applied the following filters to obtain high-quality data for training and testing our method. (1) Eukaryotic, non-plant protein sequences were only included, (2) Sequences with any ambiguous annotation like 'possible','probable', 'by similarity' and 'potential', were omitted. (3) Protein sequences localize in multiple location were removed. (4) Sequences shorter than 80 amino acids were excluded. (5). Sequences containing nonstandard amino acids such as 'X','B', and 'Z' were removed. (6) Sequences that have more than 70% similarity were removed using CD-HIT program [23]. Finally, our dataset included 1581 proteins classified into four submitochondria locations: 975 inner membrane proteins, 91 inter membrane space proteins, 238 matrix proteins and 277 outer membrane proteins.

### Features:

In this work, 239 features encoded each sequence. These features can be categorized into four groups: 60 of them are related to Composition, Centroid and Distribution features; 60 features are obtained from split amino acid composition; 88 features are extracted from protein functional groups and secondary structure information; 31 features are acquired from physico chemical properties (AA index).

### Composition, Centroid and Distribution: 

Composition, Centroid and Distribution (60 features) features were computed as described in Carr et al. 2010 [24].

### Split amino acid composition: 

The protein sequence is split into three equal parts. For each part, composition of 20 amino acid compositions was calculated. Totally, 60 feature vectors were derived from split amino acid composition.

### Frequency of functional groups:

Based on the presence of functional groups, 20 amino acids were categorized into 10 functional groups. Similarly, we categorized 20 amino acids into 7 physico-chemical groups. For each protein sequence, frequency of each amino acid group was computed and this led to 17 feature vectors [25].

### Frequency of short peptides:

From each sequence, we computed 10 residue length short peptides. Each short peptide was classified as hydrophobic, hydrophilic, neutral, polar or non-polar short peptide, and frequency of each short peptide was calculated as described in Pugalenthi et al. 2010 [25].

### Content of secondary structural element (SSE):

The overall content of helix, beta sheet and coil was computed for each sequence, Further, frequencies of 10 amino acid group and 7 physico-chemical groups at helix, sheet, and coil regions were calculated as described in Pugalenthi et al 2010 [25].

### Physicochemical properties:

As described in Kandasamy et al. 2010, we computed 31 physicochemical properties for each sequence [26].

### Classification algorithm:

SVM classification

Support Vector Machine (SVM) is a supervised machine-learning algorithm for classification and regression [27]. In this work, we used LIBSVM 2.86 package [28], which is available for downloaded from http://www.csie.ntu.edu.tw/cjlin/libsvm/. Radial Basis Function (RBF) was selected as the kernel function for the training process. The optimal value for C (penalty constant) and g (width parameter) parameters was determined using a grid search approach.

### Feature selection:

We used Information gain approach to select subset of features that play prominent role in the classification [29].

### Evaluation Parameter

We quantify prediction performance using four parameters sensitivity, specificity, overall accuracy and Matthew’s correlation coefficient (MCC). These measurements are expressed in terms of true positive (TP), false negative (FN), true negative (TN), and false positive (FP).

Sensitivity = TP/(TP+FN) → equation 1

Specificity = TN/(TN+FP) → equation 2

Accuracy = (TP + TN)/(TP + FP + TN+ FN) → equation 3

### Matthews's Correlation Coefficient (MCC): 

It is the statistical parameter to assess the quality of prediction and to take care of the unbalancing in data. It ranges from –1 ≤ MCC ≤ 1. A value of MCC = 1 indicates the best possible prediction while MCC = -1 indicates the worst possible prediction (or anti- correlation). Finally, MCC = 0 would be expected for a random prediction scheme.

MCC = (TP*TN – FP*FN)/√(TP+FN)(TP+FP)(TN+FP)(TN+FN) → equation 4

### Area under the Curve (AUC):

The Receiver Operating Curve (ROC) provides a threshold independent measure. The ROC is a plot between the true positive rate (TP/TP+FN) and the false positive rate (FP/FP+TN).

## Results and Discussion:

**Figure 2:**
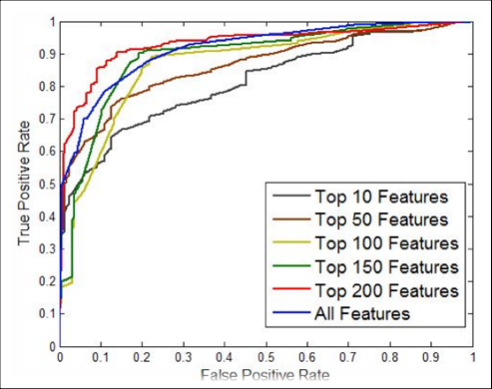
ROC curves of multi-class SVM classification (4 subclasses) on test dataset

In multi-class classification, we trained our SVM model on the training dataset containing 600 inner membrane proteins, 80 inter membrane space, 180 matrix and180 outer membrane proteins. SubMitoLoc achieved 86.05% training accuracy using all features. We carried out feature selection to identify the subset of features that play role in the classification. We selected five subsets of features that include top 200, 150, 100, 50 and 10 features, respectively. The performance of each feature subset is given in (Table 1 and Table 2). We tested our model with a test dataset of 541 proteins consists of 375 inner membrane proteins, 11 inter membrane space proteins, 58 matrix proteins and 97 outer membrane proteins. Using top 150 features, our model obtained an overall accuracy of 88.17%. The sensitivity for the proposed approach for inner membrane proteins is 90.67%, for inter membrane space is 81.82%, for matrix proteins is 84.48%, and for outer membranes is 84.54%. These results indicate that the top 200 features from Info-gain are capable of extracting more information about a primary sequence and obtaining a better prediction performance. The overall accuracy was increased from 85% to 88% when features were reduced to 200. The area under curve for all features was 0.91 and for the top 200 features was 0.93, respectively (Figure 2). This shows that our method selected more informative features and eliminated less contributing features without any drop in the accuracy. When the features were further reduced to 100, we obtained 84.47% accuracy. The accuracy decreased by only 2% when compared to the accuracy of all 239 features. Our method produced 72% accuracy with just 10 features. The results suggest that the Info-gain feature selection approach selected useful features that have significant effect in the mitochondrial and non-mitochondrial protein sequence prediction.

## Conclusions:

It is of interest to describe the identification of mitochondrial sub cellular locations of proteins from sequence derived properties using Support Vector Machine (SVM) abbreviated as SubmitoLo in this report. The model distinguishes proteins among four mitochondrial sub-cellular locations: mitochondrial inner membrane, mitochondrial outer membrane, mitochondrial inter membrane space and mitochondrial matrix with 88.6% accuracy under cross validation. The model is useful to assign mitochondrial sub cellular locations to several uncharacterized proteins to help in research and development through prediction data. We plan to implement a prediction tool in future for this purpose.

## Figures and Tables

**Table 1:** The predictive results on the 1581 mitochondrial proteins (4 classes) using SVM model.

Features	Test accuracy (%)	10 fold CV accuracy (%)
10	72.08	72.12
50	79.11	77.21
100	84.47	84.32
150	85.39	85.86
200	88.17	88.56
All features	85.02	86.05

**Table 2:** Individual accuracies for each location using top 200 features (Info-Gain) of SVM model.

4 class	Sensitivity (%)	Specificity (%)
Inner membrane	90.67	82.53
Inter membrane space	81.82	88.3
Matrix proteins	84.48	89.23
Outer membrane	84.54	88.96
